# New and Safe Treatment of Food Impacted in the Esophagus: A Single Center Experience of 100 Consecutive Cases

**DOI:** 10.1155/2013/142703

**Published:** 2013-11-18

**Authors:** Muhammad Shafique, Sheraz Yaqub, Erik S. Lie, Vegard Dahl, Frode Olsbø, Ola Røkke

**Affiliations:** ^1^Department of Gastroenterological Surgery, Akershus University Hospital, 1478 Lørenskog, Norway; ^2^Department of Gastroenterological Surgery, Oslo University Hospital, 0316 Oslo, Norway; ^3^Department of Ear-Nose-Throat, Akershus University Hospital, 1470 Lørenskog, Norway; ^4^Faculty of Medicine, University of Oslo, 0316 Oslo, Norway; ^5^Department of Anesthesiology, Akershus University Hospital, 1470 Lørenskog, Norway; ^6^Department of Thoracic and Vascular Surgery, Akershus University Hospital, 1470 Lørenskog, Norway

## Abstract

*Aim*. Large food bits can get stuck in the esophagus and must be removed by endoscopy. In some cases, this can be difficult or unsafe. We describe a new and safe treatment for such patients. *Materials and Methods*. 100 consecutive patients were referred to Akershus University Hospital with impacted food in the esophagus. In 36 patients (36%), the food passed spontaneously. In 59 (92%) of the remaining 64 patients, the food was removed by endoscopic intervention. In the last five patients, endoscopic removal was judged difficult or unsafe. These patients received the new treatment: one capsule Creon 10000 IU dissolved in 30 mL of Coca-Cola administered by a nasooesophageal tube four times daily for 2-3 days. *Results*. Of the 59 patients treated with endoscopic procedure, complications occurred in four (7%): three bleedings and one perforation of the esophagus. In five patients treated with Coca-Cola and Creon, the food had either passed or was soft after 2-3 days and could easily be removed. *Conclusion*. The treatment of choice of impacted food in the esophagus is endoscopic removal. In cases where this is difficult, we recommend treatment with Coca-Cola and Creon for 2-3 days before complications occur.

## 1. Introduction

Food may sometimes get stuck in the esophagus, most frequently during meals containing meat. Usually the diagnosis is easy; the patient notices that the food is impacted and experiences dysphagia, regurgitation, pain, and vomiting, which makes it impossible to continue the meal.

Impacted food may eventually pass spontaneously to the stomach without further actions, but sometimes it requires treatment in hospitals. The recommended method is to perform an upper endoscopy, and the impacted food is either extracted orally or pushed down into the stomach. In cases where food is impacted in the upper part of esophagus, rigid endoscopes may be used in experienced hands. However, removal can sometimes be difficult due to large size or sticky quality of the food bolus or local conditions of the esophageal wall, like stenosis due to edema or stricture. In such circumstances, endoscopic procedures may lead to complications, of which the most feared and serious is perforation of the esophagus. This is a potential fatal complication. At Akershus University Hospital, we have adopted a new treatment method in such situations. The method was developed during an attempt to endoscopically remove a voluminous schnitzel stuck in the esophagus without success [1]. In this paper, we present the outcomes for 100 patients with impacted food in the esophagus, and describe the new cocktail that can dissolve impacted meat in a gentle and safe way.

## 2. Material and Method

The study consists of 100 consecutive patients, 64 men and 36 women, with median age 49.9 years (8–92 years) referred to Akershus University Hospital from February 2009 to May 2012 with impacted food in the esophagus. The incidence of esophageal food impaction varied according to the seasons. Most patients were admitted during the Norwegian winter months of December, January, and February (*n* = 37) (Table 1). 

22 patients had conditions that predisposed for foodimpaction: hiatal hernia with esophagitis (*n* = 10), esophageal stenosis (*n* = 4), neurological disease (*n* = 4), Schatzkiring (*n* = 2), achalasia (*n* = 1), and eosinophil esophagitis (*n* = 1). 13 patients reported previous episodes of food impaction. Food impaction most often occurred after meals containing different kinds of meat (*n* = 86): piece of meat (*n* = 41), spare-rib (*n* = 9), some with bones, beef (*n* = 10), chicken (*n* = 9), sausage (*n* = 8), fish (*n* = 5), duck (*n* = 2), schnitzel (*n* = 1), and meat balls (*n* = 1). However, many other types of food and substances were also impacted like potato (*n* = 1), pizza (*n* = 1), garlic (*n* = 1), tablets (*n* = 1), peanuts (*n* = 1), apple (*n* = 2), berries/fruit (*n* = 1), pea (*n* = 1), piece of glass (*n* = 1), metal object (*n* = 1), and food ingested during dinner not further specified (*n* = 3). 

In approximately 1/3 of the patients (36 patients) the food passed spontaneously during transport to hospital or shortly after arriving at the hospital. Some of these received a laxative remedy (Duphalac mixture). Spontaneous passage of food was confirmed by endoscopy in 11 (30, 6%) of these patients. In the remaining 25 (69, 4%), the patients reported subjective passage and were able to drink and eat. 

**Figure 1:**
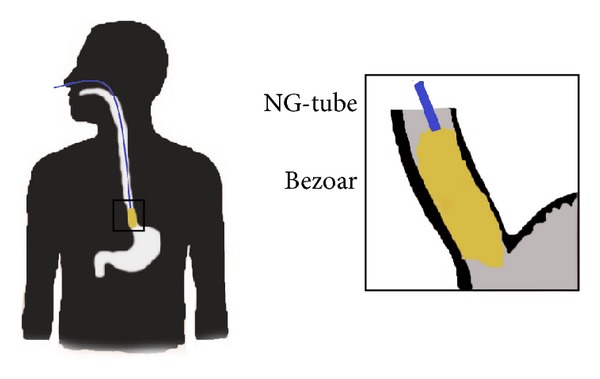
Illustration of tube placement for administration of the dissolution cocktail.

**Figure 2:**
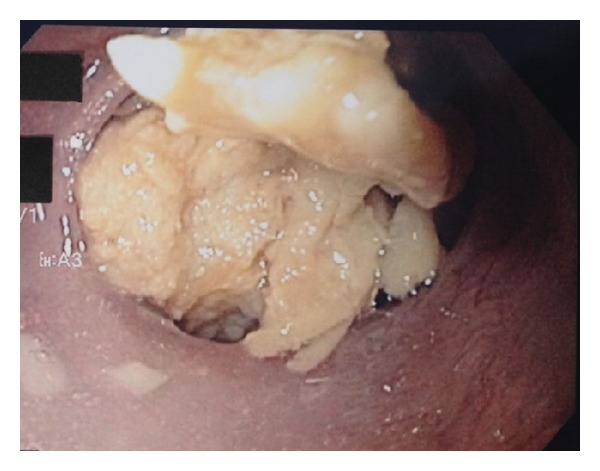
Food impacted in the distal esophagus.

In 64 patients, impacted food was diagnosed by upper endoscopy (Figure 2) and removed in 59 of them (92%). In the other five patients, the endoscopic procedure was timeconsuming and considered unsafe. These patients were treated with a new dissolving solution which consists of one capsule of pancreas digestive enzymes: Creon 10000 IE dissolved in 30 mL of Coca-Cola. This cocktail was installed four times a day for two to three consecutive days through a nasoesophageal tube where the tip of the tube was positioned oral/into the impacted food (Figure 1). The treatment was given with the patient being in a sitting position. The patients could not swallow and hence were given intravenous fluids. 

## 3. Results

Characteristics of the patients treated for impacted food and the patients where the food passed spontaneously are shown in Table 2. The age, predisposition for food impaction, and the number of patients with previous episodes of impacted food were similar in the two groups. Food impaction occurred more often in men than in women, and spontaneous passage of food was more likely in women than in men (*P* = 0.029). Spontaneous passage of food did occur in foods that needed treatment, like meat, spare-rib, beef, chicken, and sausage, but most of these impacted food materials needed treatment. However, foods like pizza (*n* = 1), fish (*n* = 5), peanut (*n* = 1), apple (*n* = 2), berries/fruit (*n* = 1), pea (*n* = 1) did pass spontaneously in this series, as the two foreign bodies (piece of glass (*n* = 1) and metal object (*n* = 1)).

 64 patients needed treatment. In these patients, the impacted food was detected in the upper (11%), middle (14%), or lower (75%) third of the esophagus. In 42 patients (65.6%), food was pushed down into the stomach, while the food was extracted orally in 17 patients (26.6%). In five patients (8%), attempt of endoscopic removal was timeconsuming and unpleasant for the patient, and these were instead treated with the Coca-Cola-Creon-cocktail. At follow-up with upper endoscopy three days after the start of the treatment the food had passed completely in three patients, or had become soft and fragmented, and the remaining parts could easily be pushed down into the stomach in the other two. Characteristics of the patients treated with endoscopic removal and the Coca-Cola-Creon-cocktail are shown in Table 3. The groups were similar with regard to age, gender, and type of impacted food. Complications occurred in four patients (7%) after endoscopic removal: three minor complications: grade I and II (2), two minor bleedings and one bleeding in need of blood transfusion in patients using anticoagulation, and one major complication: grade IIIb: esophageal perforation, treated with acute thoracolaparotomy and suture of the perforation. There were no complications related to the Coca-Cola-Creon-coctail treatment. 

## 4. Statistics

Continuous variables are presented as median (minimum-maximum) in tables and text. Student's *t*-test was used to test differences between means. Pearson's chi-squared test was used to test differences between groups.

## 5. Discussion

It is not uncommon that food gets impacted in the esophagus. In the present study, the food passed spontaneously or with treatment with laxative (Duphalac) in about 1/3 of the patients. This is a harmless outpatient treatment, as long as the patient is able to swallow saliva. Food like fish and berries/fruits was likely to pass without treatment. In the remaining 2/3 of patients, food had to be removed by intervention. 

Endoscopic removal is the gold standard, and in most cases this intervention is successful without complications. However, endoscopic intervention may require sedation or general anesthesia. The procedure can be timeconsuming and may require greater use of power than desired. In such situations, there is a high risk of complications, such as esophageal perforation and bleeding induced by manipulation of the esophageal wall with endoscopic instruments. Perforation is feared and is a potential life-threatening complication, which requires immediate recognition and treatment. In our series, esophageal perforation occurred in one patient and was immediately surgically treated by thoracolaparotomy and suture of the ruptured esophagus. This patient experienced late complications and was reoperated twice due to low grade periostal infection in a costa. Three other patients experienced complications with bleeding. These complications may have been avoided if endoscopic procedure had been aborted earlier and converted to the new dissolution method.

The method of dissolution of impacted meat was developed under the care of a patient who during a meal got a schnitzel jammed in the distal 10 centimeters of the esophagus, previously described in detail [1]. Repeated sessions of endoscopic treatment were unsuccessful. Previous studies of patients with bezoar in the stomach had shown a possibility with chemical fragmentation and/or resolution with Coca-Cola [2–9]. Coca-Cola contains sodium bicarbonate (NaHCO_3_), which acts as an expectorant/mucolytic agent. Furthermore, carbon dioxide gas (CO_2_) penetrates the food and the low pH of 2.6 dissolves fibers in the food. Creon 10 000 consists of animal digestive enzymes, which contain 8.000 IU of amylase, 10.000 IU of lipase, and 600 IU of protease that despite the low pH of the Coca-Cola can contribute to resolving leftovers. Since there was considerable danger of perforation of esophagus in this patient, we chose a resolution attempt. We installed a mixture of 30 mL of Coca-Cola and the contents of one capsule Creon 10 000 in the bezoar four times daily for four days through an esophageal tube with the tip located just above the impacted food. At endoscopy five days after the start of the treatment, the esophagus was clean, with no signs of food. This inspired us to continue this dissolution treatment as an option in difficult cases. 

The presence of bones in the foods may increase the risk of perforation, both at impaction and on extraction. In some cases, such bones are actually seen stuck into the esophageal wall at endoscopy and should be extracted, and the patients should be observed for symptoms of esophageal perforation. In the present series, no bones were actually identified at endoscopy in the impacted food, and only a few patients reported possible bone ingestion (spare-ribingestion). But the possibility that the food bolus could contain bone should be kept in mind by the endoscopist so that removal is done with care. If no bone is seen stuck into the oesophageal wall at endoscopy, we think that the dissolution method is suitable also for foods containing bones. Until now, five patients have been successfully treated with this method without complications after unsuccessful endoscopic removal. 

## 6. Conclusions

Dissolving treatment of impacted food in the esophagus with Creon dissolved in Coca-Cola is a promising method in cases where endoscopic removal is unsuccessful. In our experience with five patients, the method was safe and effective. We suggest that this method should be considered in cases where endoscopic removal of impacted food is difficult, before complications occur. 

## 7. Conflict of Interests

The authors declare that there is no conflict of interests regarding the publication of this paper.

## Figures and Tables

**Table 1:** 

Season	n
Winter (December, January, February)	37
Spring (March, April, May)	18
Summer (June, July, August)	25
Autumn (September, October, November)	20

Sum	100

**Table 2:** 100 patients admitted to hospital with impacted food in the oesophagus.

	Impacted food in need of treatment *n* = 64	Spontaneous passage *n* = 36	*P*
Age (years) median (min–max)	50,8 (8–92)	47,0 (9–90)	0,781
Gender			0,029
Male	46 (71,9%)	18 (50%)	
Female	18 (28,1%)	18 (50%)	
Predisposition for food impactions			0,792
None	49 (76,6%)	29 (80,6%)	
Hiatal hernia/esophagitis	7 (10,9%)	3 (8,3%)	
Stenosis	2 (3,1%)	2 (5,6%)	
Schatzki ring	2 (3,1%)	0	
Achalasia	1 (1,6%)	0	
Eosinophil esophagitis	1 (1,6%)	0	
Neurological conditions	2 (3,1%)	2 (5,6%)	
Previous episodes of food impaction	9 (14,1%)	4 (11,1%)	0,674
Food type			0,013
Meat	29 (45,3%)	11 (30,6%)	
Spare-rib	8 (12,5%)	0	
Beef	7 (10,9%)	3 (8,3%)	
Chicken	6 (9,4%)	3 (8,3%)	
Sausage	5 (7,8%)	3 (8,3%)	
Others	9 (14,1%)	16 (44,4%)	

**Table 3:** 64 patients treated for impacted food in the oesophagus.

	Endoscopic removal *n* = 59	Coca-Cola + Creon *n* = 5	*P*
Age (years) (mean ± SD)	50,5 (8–91)	52,8 (23–92)	0,401
Gender Male Female	42 (71,2%) 17 (28,8%)	4 (80%) 1 (20%)	0,674
Food type Meat Spare-rib Beef Chicken Sausage Others	28 (47,5%) 7 (11,9%) 7 (11,9%) 4 (6,8%) 5 (8,5%) 8 (13,6%)	1 (20%) 1 (20%) 0 2 (40%) 0 1 (20%)	0,180
Complications Oesophageal perforation	1	0	0,548
Bleeding in need of transfusion	1	0	
Minor bleeding	2	0	
